# Nanotechnology-Enabled Combination Therapies and Diagnostic Innovation: An Integrative Overview of Recent Advances

**DOI:** 10.3390/pharmaceutics17121620

**Published:** 2025-12-17

**Authors:** Renato Sonchini Gonçalves

**Affiliations:** Laboratory of Chemistry of Natural Products, Department of Chemistry, Federal University of Maranhão (UFMA), São Luís 65080-805, Brazil; renato.sg@ufma.br; Tel.: +55-98-985-149-235

## 1. Introduction

Noncommunicable diseases (NCDs) and other high-impact health challenges continue to impose a substantial and persistent burden on global public health [1,2]. According to the World Health Organization, NCDs have dominated the leading causes of mortality for the past two decades, reflecting the complexity of conditions such as cancer, chronic inflammatory disorders, metabolic dysfunctions, and pathogen-associated complications. This scenario underscores the urgency of developing innovative therapeutic and diagnostic strategies that are not only more effective and safer but also mechanistically robust enough to address the multifactorial nature of these diseases.

Within this context, nanotechnology has emerged as a transformative platform capable of reshaping current therapeutic paradigms [3,4,5,6]. Advances in nanoscale engineering now enable the design of combination therapies that integrate multiple mechanisms of action within a single system, improve drug solubility and bioavailability, optimize spatial and temporal biodistribution, and offer opportunities for real-time diagnostic monitoring. These capabilities have expanded the scope and effectiveness of conventional pharmacological interventions, supporting a new generation of precision therapeutics tailored to the biological heterogeneity characteristic of NCDs.

In parallel with these technological advances, the awarding of the 2025 Nobel Prize in Chemistry to Susumu Kitagawa, Richard Robson, and Omar M. Yaghi for the development of metal–organic frameworks (MOFs) has further emphasized the foundational impact of reticular chemistry on modern nanomedicine [7,8,9,10,11]. The modularity, high porosity, and tunable chemical environments inherent to MOFs—and particularly their nanoscale counterparts (nMOFs) [8,9,11]—provide an unprecedented degree of structural control for the creation of multifunctional platforms. These properties enable the rational integration of therapeutic payloads, targeting ligands, and photoactive or radioreactive components, reinforcing the strategic potential of MOF-based architectures for combination therapy, controlled drug delivery, and activation through optical or ionizing radiation. This recognition aligns closely with the emerging trends highlighted in this Special Issue, where framework-based materials increasingly serve as central elements in advanced therapeutic strategies.

**Figure 1:**
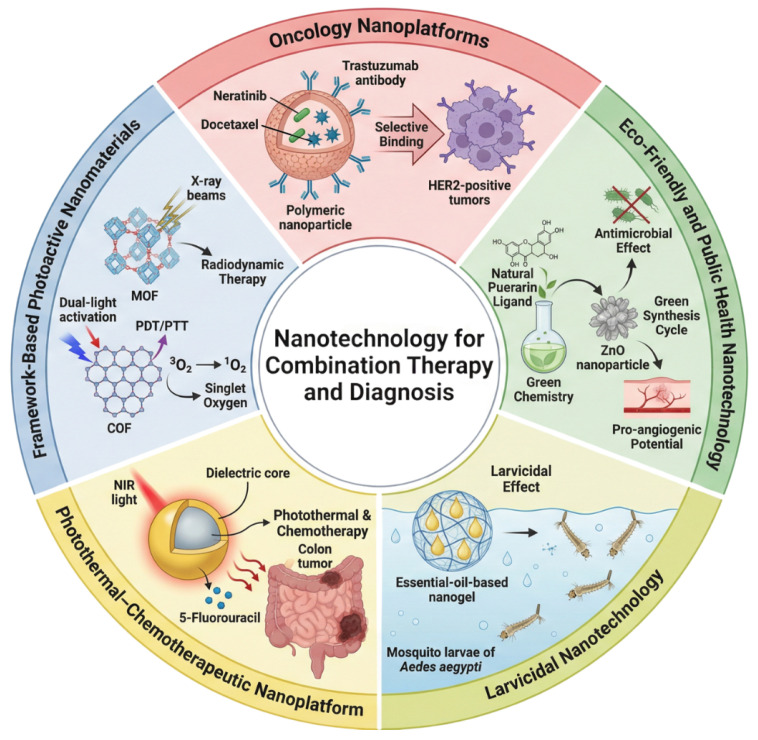
Conceptual overview of the nanotechnological strategies highlighted in this Special Issue, illustrating the integration of multifunctional oncological nanoplatforms, photoactive framework-based materials (MOFs and COFs), eco-friendly nanotechnologies, and natural-product nanogels for vector control. The diagram emphasizes how nanoscale engineering enables targeted drug delivery, chemophotothermal and radiodynamic activation, green-synthesized antimicrobial platforms, and sustainable larvicidal formulations. Collectively, these domains represent the convergent landscape of advanced nanotechnology for combination therapy and diagnostic innovation.

The intersection between epidemiological challenges, chemical innovation, and nanoscale engineering defines the landscape in which the contributions of this Special Issue, Advanced Nanotechnology for Combination Therapy and Diagnosis, are situated [3,4,5,6]. Collectively, the studies published here illustrate how nanotechnology continues to evolve as a key enabler of integrative and synergistic therapeutic approaches, providing versatile and adaptive solutions for some of the most pressing biomedical challenges of our time. A conceptual overview of these nanotechnological strategies is presented in Figure 1.

## 2. Overview of Published Work

The contributions gathered in this Special Issue collectively demonstrate the scientific maturity and multidimensional scope of contemporary nanotechnology applied to combination therapy and diagnostic innovation. Across diverse pathological contexts—including oncology, infectious diseases, and vector control—the studies illustrate how rational nanoscale engineering enhances therapeutic potency, improves biological selectivity, and expands the range of functional modalities available for clinical intervention.

A strong focus on oncological applications emerges through nanoplatforms designed to integrate pharmacological synergy, molecular targeting, and photoactivated mechanisms. One study reports a dual-drug polymeric nanoparticle co-loaded with neratinib and docetaxel and surface-functionalized with trastuzumab, providing a highly specific therapeutic approach for HER2-positive breast cancer [12]. By combining ligand-directed recognition with hydrophobic drug encapsulation, this system exemplifies how nanocarriers can overcome tumor heterogeneity and promote enhanced intracellular drug delivery [5,6]. Complementing this strategy, another investigation employs a chemophotothermal modality in which branched gold nanoshells potentiate the cytotoxicity of 5-fluorouracil under photothermal activation [13]. This design highlights the capability of plasmonic nanostructures to circumvent resistance mechanisms, amplify chemotherapeutic effects, and induce significant tumor reduction in a colorectal cancer xenograft model.

Beyond cancer therapy, the Special Issue includes contributions that underscore the intersection between nanotechnology, sustainability, and antimicrobial or vector-control strategies. The green synthesis of zinc oxide nanoparticles using puerarin demonstrates how bio-derived ligands govern nanoparticle morphology, stability, and functional behavior, yielding systems with antimicrobial activity, angiogenic modulation, and a favorable in ovo safety profile [14]. These findings are consistent with broader advances in green ZnO-based nanotechnology for biomedical and antimicrobial applications [15]. In parallel, the development of an essential-oil-based nanogel targeting *Aedes aegypti* larvae reveals how natural-product nanostructures can support environmentally responsible approaches to vector management, addressing a persistent public health challenge in arbovirus-endemic regions [16], in line with emerging eco-friendly nanobiocides for mosquito control [17,18].

Complementing these research articles, two reviews provide an in-depth examination of emerging frontiers in photoactive framework-based nanomaterials. The first discusses porphyrin-based nMOFs designed for radiodynamic therapy, emphasizing their capacity for deep-tissue activation through X-ray excitation, a key advancement over conventional photodynamic approaches limited by shallow light penetration [19]. The second review focuses on porphyrin-based covalent organic frameworks (COFs) and highlights how their tunable porosity, extended π-conjugation, and modular architecture support synergistic photodynamic and photothermal therapeutic modalities [20]. Together, these reviews demonstrate how reticular chemistry—recently recognized by the 2025 Nobel Prize in Chemistry—continues to drive innovation in multifunctional nanoplatform design [7,8,9,10,11].

Collectively, the works presented in this Special Issue reflect a rapidly progressing field in which chemical design, supramolecular structuring, environmentally conscious synthesis, and photonic or radiative activation converge to advance the frontiers of combination therapy and diagnostic nanotechnology [3,4,5,6,12,13,14,16,19,20]. The diversity of materials and mechanisms represented here underscores the ongoing transformation of nanomedicine into a highly integrated and translational scientific discipline.

## 3. Future Perspectives

The perspectives emerging from the contributions in this Special Issue highlight a rapidly evolving landscape in which nanotechnology continues to redefine the conceptual and practical boundaries of combination therapy and diagnostic innovation [3,4,5,6,12,13,14,16,19,20]. Over the coming years, advances are expected to be driven by deeper integration of molecular design, supramolecular organization, stimuli-responsive behaviors, and predictive computational tools capable of guiding the rational engineering of multifunctional nanoplatforms [3,4,5,6].

One of the most promising trajectories involves the development of adaptive nanosystems capable of integrating multiple therapeutic and diagnostic modalities within a single architecture [3,4,5,6,12,13,14,16]. The convergence of pharmacological co-delivery, molecular targeting, photonic or radiative activation, and controlled release mechanisms will be increasingly supported by high-resolution analytical methods and machine learning–assisted optimization [4,5,6]. These strategies are poised to enhance spatiotemporal precision, improve therapeutic indices, and mitigate the onset of multidrug resistance—challenges that remain central in the management of complex diseases such as cancer [5,6].

Another key direction relates to the continuous expansion of photoresponsive and radioresponsive nanostructures. The insights provided by the reviews included in this Special Issue indicate that framework-based nanomaterials, including those inspired by the principles of reticular chemistry recognized by the 2025 Nobel Prize in Chemistry, offer unparalleled opportunities for deep-tissue activation, structured energy transfer, and multimodal phototherapeutic action [7,8,9,10,11,19,20]. Future efforts may focus on hybrid activation platforms that combine optical, thermal, and ionizing stimuli, thereby enabling therapeutic modalities not achievable with conventional photosensitizers or single-mechanism approaches [8,9,11,19,20].

Sustainability will also shape the next generation of nanotechnology-enabled therapies. The works employing green-synthesized nanoparticles and essential-oil-based nanostructures demonstrate the feasibility of environmentally responsible approaches that preserve or enhance therapeutic performance [14,15,16,17,18]. Continued innovation in solvent-free synthesis routes, renewable precursors, and biodegradable nanomaterials is expected to expand the ecological compatibility of nanomedicine while supporting safer translational pathways [15,18].

Finally, accelerating clinical translation remains a key challenge and opportunity. Progress in regulatory science, scalable manufacturing, and standardized physicochemical and toxicological characterization will be indispensable for bridging the gap between laboratory prototypes and clinically validated nanotherapeutics [4,5,6]. Integrated frameworks that account for long-term biodistribution, immunomodulatory effects, and environmental persistence will be essential for ensuring safety and reproducibility in increasingly complex multimodal nanosystems [4,6,11].

Together, these perspectives outline a future in which chemical innovation, bioengineering, materials science, and computational design converge to drive the next generation of nanotechnology-based combination therapies and diagnostic tools. As demonstrated by the contributions in this Special Issue, the field is poised to deliver transformative solutions to some of the most persistent biomedical challenges of the twenty-first century [3,4,5,6,12,13,14,16,19,20].
